# 1839. Impact of Hypoalbuminemia on Ceftriaxone Treatment Failure in Patients With *Enterobacterales* Bacteremia

**DOI:** 10.1093/ofid/ofac492.1468

**Published:** 2022-12-15

**Authors:** Evan Steere, Taryn Eubank, Sage Greenlee, Megan Cooper, Ty C Drake

**Affiliations:** Houston Methodist Hospital, Houston, Texas; Houston Methodist Hospital, Houston, Texas; Houston Methodist Hospital, Houston, Texas; Houston Methodist Hospital, Houston, Texas; Houston Methodist Willowbrook Hospital, Houston, Texas

## Abstract

**Background:**

Ceftriaxone (CRO) is a third-generation cephalosporin that is commonly prescribed due to its robust Gram-negative activity and lack of renal dose adjustments. It is also highly protein bound allowing for once daily dosing. In patients with hypoalbuminemia, however, the proportion of free drug is increased thus increasing the rate at which CRO is renally eliminated. This increased clearance could lead to lower serum concentrations and increased risk of treatment failure.

**Methods:**

We performed a retrospective, cohort study to assess the impact of hypoalbuminemia (serum albumin ≤ 2.5 g/dL) on treatment failure among patients with monomicrobial *Enterobacterales* bacteremia. Adult patients who received > 72 hours of CRO therapy for susceptible *Enterobacterales* bacteremia between May 1, 2016 and April 30, 2021 were eligible for inclusion. The primary outcome of treatment failure was a composite of inpatient mortality or escalation to an intravenous anti-pseudomonal antibiotic or ertapenem. Secondary outcomes included hospital length of stay, total duration of antibiotic therapy, and time to infection resolution. Baseline characteristics and outcomes were compared using R Studio (Version 4.1.0) in a 1:1 propensity-matched cohort.

**Results:**

After propensity score matching, 142 patients were included in each study group. The most common organisms in our cohort were *Escherichia coli* (71.5%), *Klebsiella pneumoniae* (14.1%), and *Proteus mirabilis* (5.3%). The most frequently implicated source of infection was the urinary tract (71.5%). Interestingly, treatment failure was numerically higher in the hypoalbuminemia group but was not statistically significant (12.0% vs. 7.7%, p = 0.23). All secondary outcomes were similar between groups. Among the subgroup of patients admitted to the ICU at baseline, the difference in treatment failure was even greater between patients with hypoalbuminemia and those without (23.3% vs. 7.5%, *P* = 0.07).

**Conclusion:**

Hypoalbuminemia did not appear to have a significant impact on clinical outcomes among patients with *Enterobacterales* bacteremia treated with CRO. However, critically ill patients may be subject to higher incidence of treatment failure in the presence of hypoalbuminemia.

**Disclosures:**

**All Authors**: No reported disclosures.

